# Schottky Barrier Height Tuning via the Dopant Segregation Technique through Low-Temperature Microwave Annealing

**DOI:** 10.3390/ma9050315

**Published:** 2016-04-27

**Authors:** Chaochao Fu, Xiangbiao Zhou, Yan Wang, Peng Xu, Ming Xu, Dongping Wu, Jun Luo, Chao Zhao, Shi-Li Zhang

**Affiliations:** 1 State Key Laboratory of ASIC and System, Fudan University, Shanghai 200433, China; 12110720010@fudan.edu.cn (C.F.); zhouxiangbiao@huawei.com (X.Z.); 14210720099@fudan.edu.cn (Y.W.); 11110720013@fudan.edu.cn (P.X.); xu.ming@zte.com.cn (M.X.); 2 Key Laboratory of Microelectronic Devices and Integrated Technology, Institute of Microelectronics, Chinese Academy of Science, Beijing 100029, China; luojun@ime.ac.cn (J.L.); zhaochao@ime.ac.cn (C.Z.); 3 Solid-State Electronics, The Ångström Laboratory, Uppsala University, P.O. Box 534, Uppsala 75121, Sweden; shili.zhang@angstrom.uu.se

**Keywords:** microwave annealing, Schottky barrier height, MOSFETs, dopant segregation, low temperature, diode

## Abstract

The Schottky junction source/drain structure has great potential to replace the traditional p/n junction source/drain structure of the future ultra-scaled metal-oxide-semiconductor field effect transistors (MOSFETs), as it can form ultimately shallow junctions. However, the effective Schottky barrier height (SBH) of the Schottky junction needs to be tuned to be lower than 100 meV in order to obtain a high driving current. In this paper, microwave annealing is employed to modify the effective SBH of NiSi on Si via boron or arsenic dopant segregation. The barrier height decreased from 0.4–0.7 eV to 0.2–0.1 eV for both conduction polarities by annealing below 400 °C. Compared with the required temperature in traditional rapid thermal annealing, the temperature demanded in microwave annealing is ~60 °C lower, and the mechanisms of this observation are briefly discussed. Microwave annealing is hence of high interest to future semiconductor processing owing to its unique capability of forming the metal/semiconductor contact at a remarkably lower temperature.

## 1. Introduction

The tuning of Schottky barrier height (SBH) between metal silicide and underlying Si by the dopant segregation (DS) technique has recently been extensively explored for the development of integrated circuits of advanced technology nodes. The barrier height of the junction in the actual metal-semiconductor interface is deemed effective SBH, for which the influence of the electric field is taken into account [1]. Ultra-low contact resistivity between Ni_1-x_Pt_x_Si and Si at the level of 6–7 × 10^−9^ Ohm∙cm^−2^, which is highly desirable for the 3-D metal-oxide-semiconductor field effect transistor (MOSFET) structures for the 15-nm node and beyond, has been reported with SBH tuning with the DS technique [2]. Meanwhile, a metal silicide Schottky junction source/drain (S/D) structure with low effective SBHs tuned by DS has also attracted substantial research interest [3,4], since it has great potential to replace the conventional shallow p/n junction S/D in the future ultra-scaled MOSFETs, which require extremely shallow and low series resistance S/D regions.

The effective SBH is required to be lower than 100 meV in order to obtain a high driving current for the application of the Schottky junction S/D in the scaled MOSFETs [4,5]. To introduce dopants into the metal silicide/Si interface and hence tune the effective SBH towards 100 meV, mainly two DS schemes have been explored: silicide-induced dopant segregation (SIDS) [6,7] and silicide as a diffusion source (SADS) [6,7,8]. In both cases, the dopants are driven and finally segregate at the silicide/Si interface by a thermal treatment such as the rapid thermal annealing (RTA) process. The RTA temperature has reportedly been above 500 °C in order to effectively tune SBH to the saturated level [6,7,8].

The application of microwave annealing (MWA) in semiconductor processing has recently gained increasing attention due to the unique features related to the MWA process. MWA has been reported to be able to form metal silicide [9,10,11,12] and activate dopants [11,13] at a significantly lower temperature compared with the conventional RTA process. The temperature differences were reported to be around 100 °C [9,10,11] or even close to 200 °C [11,13]. In this work, we experimentally demonstrate SBH tuning with the DS technique with a low-temperature MWA process and explore the related mechanisms. The SIDS scheme is adopted in the experiments due to its effectiveness [6,7] in SBH tuning and relatively simpler process flow compared with the SADS scheme.

## 2. Experimental Section

**Figure 1:**
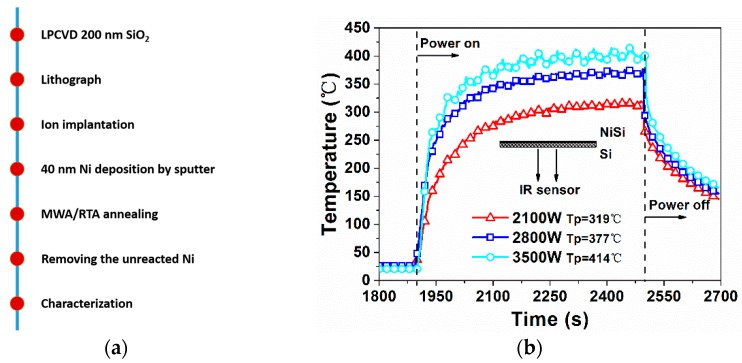
(**a**) Process flow; (**b**) temperature profiles of the MWA processes performed at different microwave powers.

Both p-type and n-type 4-inch (100) epitaxial wafers were used as the substrates. The thickness of the 1–10 Ω∙cm epitaxial layer was 5 μm. After the deposition of a 200-nm-thick SiO_2_ layer via low-pressure chemical vapor deposition, lithography and dry etch of the SiO_2_ layer were conducted to form a circular hole with a diameter of 100 μm. Boron (B) with a dose of 1 × 10^15^ cm^−2^ at 1 keV and arsenic (As) with a dose of 1 × 10^15^ cm^−2^ at 7 keV were then implanted into the n-type and p-type substrates, respectively. After removal of the photoresist, a 40-nm Ni film was deposited via sputter, preceded by a wet cleaning step with hydrofluoric acid to remove the remaining oxide on the Si surface in the hole. The wafers were then sliced into square-shaped samples with a size of about 25 mm × 25 mm, followed by a drive-in anneal via MWA or RTA for various durations and at various temperatures in a N_2_ atmosphere (see Table 1). The schematic description of the process flow is illustrated in Figure 1a.

**Figure A1:**
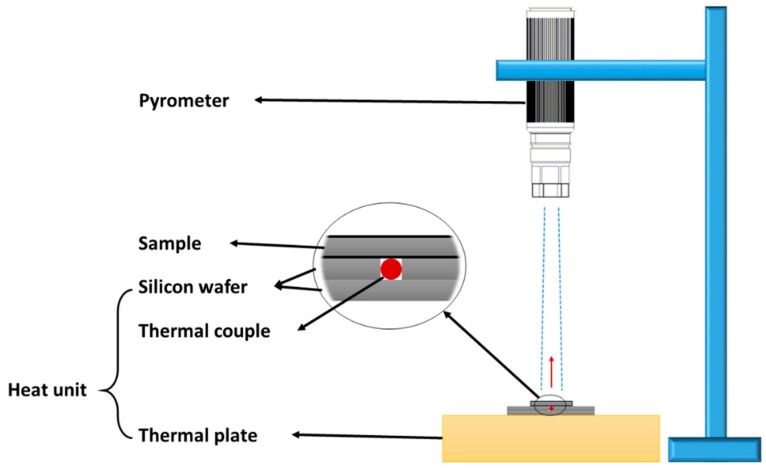
Schematic illustration of the temperature calibration system.

**Figure A2:**
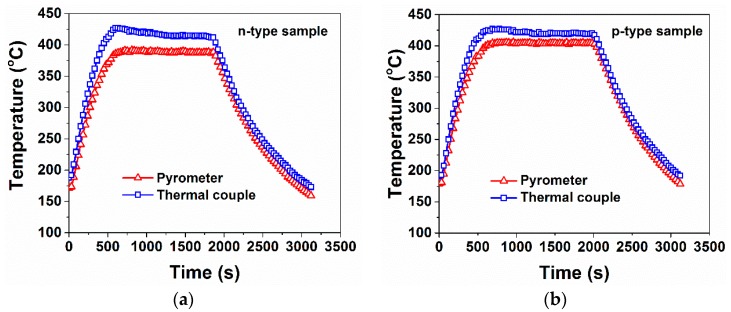
Temperature profile of pyrometer and thermal couple of (**a**) the n-type sample and (**b**) the p-type sample.

**Figure A3:**
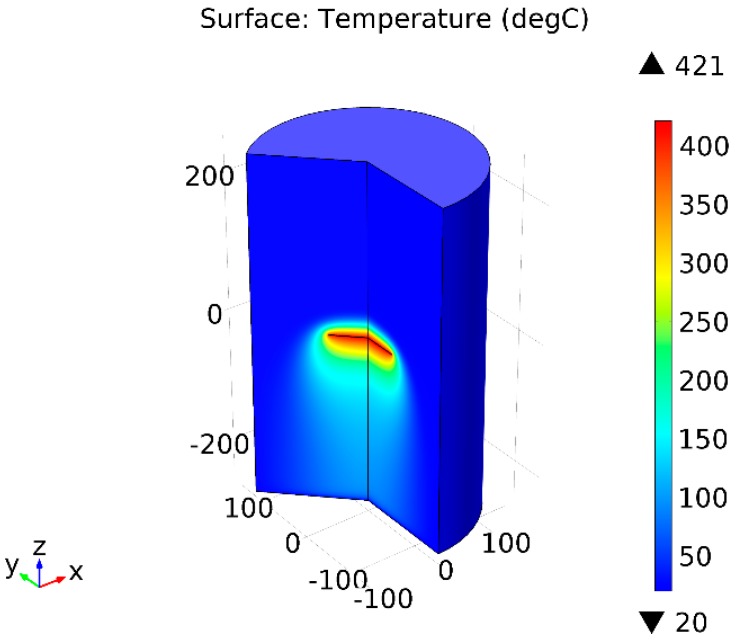
The simulation result of heat transfer of the chamber and the wafer.

**Figure A4:**
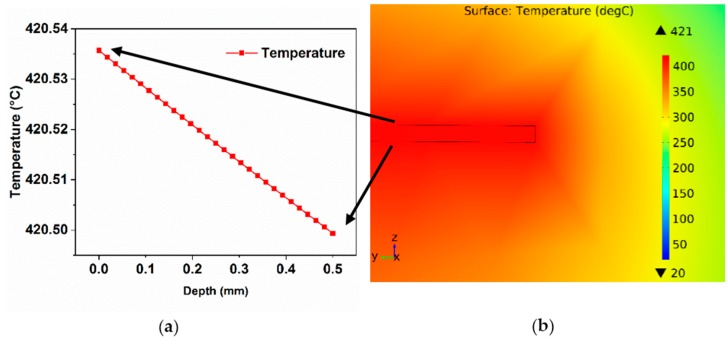
(**a**) The temperature *versus* the depth from the surface of the wafer; (**b**) The zoomed cross-sectional view of the temperature profile of the wafer.

The MWA was carried out in a DSGI (DSG Incorporation, Santa Clara, CA, USA) octagonal MWA chamber at a frequency of 5.8 GHz. Vertically stacked wafers are supported by three quartz rods inside a quartz chamber. The samples were placed in the middle of the chamber where the electromagnetic field is most uniform. The sample temperature was monitored directly using a Raytek (Santa Cruz, CA, USA) XR infrared pyrometer, which is sitting at the bottom of the MWA chamber and facing the backside of the wafer. The measured temperature is supposed to reflect the temperature of the backside of the bulk Si. Calibration work of experimental (Figure A1 and Figure A2) and simulation (Figure A3 and Figure A4) was done to understand the error of the temperature measurement of the MWA facility. The infrared pyrometer was found to underestimate the temperature of the samples, and the deviation is shown to be no more than 43 °C and 30 °C for the n-type and p-type samples in this work (Figure A2), respectively. A simple simulation of heat transfer was also carried out to study the temperature difference that may exists between the two sides of the wafer. The detail of the calibration and simulation is depicted in Appendix A.

The temperature profiles and the corresponding peak temperatures (Tp) of the MWA-treated samples are shown in Figure 1b. For comparison with the MWA samples and other published results [6,7,8], RTA with durations of both 600 and 30 s were performed. After annealing, the unreacted Ni was selectively removed with a piranha solution, which is a mixture of sulfuric acid and hydrogen peroxide.

## 3. Results and Discussion

**Figure 2:**
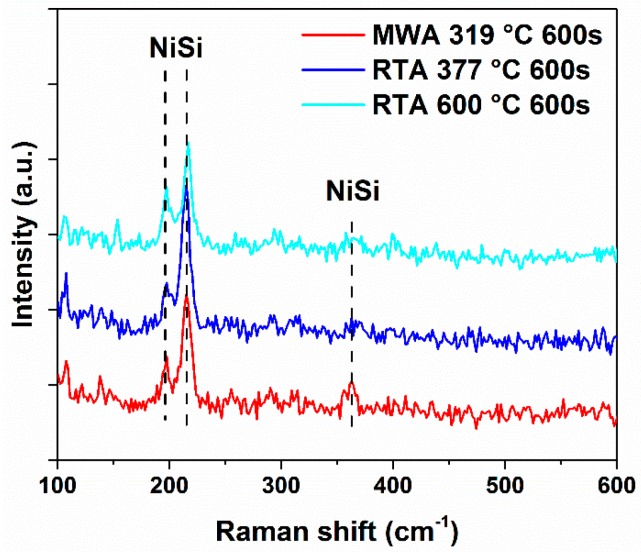
Raman spectra of the n-type samples.

The Raman spectra of RTA 377 °C, RTA 600 °C, and MWA 319 °C annealed n-type samples were measured and are shown in Figure 2. The monosilicide (NiSi) is formed under all the three annealing conditions according to the appearance of the peaks of NiSi reported in the literature [14]. No obvious Ni_2_Si peaks [15] are detected, indicating the transformation from Ni_2_Si to NiSi has almost been accomplished. Additionally, there are no peaks of NiSi_2_ [16] observed in the spectrum of the RTA 600 °C sample, for the temperature is much lower than 750 °C, which is the transition point of NiSi to NiSi_2_ [17]. Since the samples with the highest temperature and the lowest temperature all form nickel monosilicide, it is reasonable to conclude that the samples of the medium temperatures have also formed nickel monosilicide.

**Figure 3:**
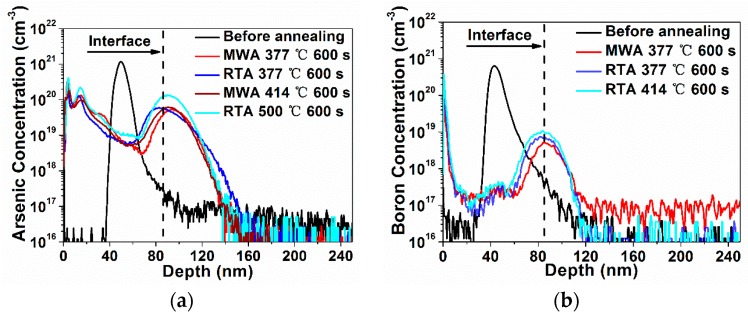
Secondary ion mass spectrometry (SIMS) profiles of (**a**) As dopants on p-type substrate; and (**b**) B dopants on n-type substrate before and after annealing.

As shown in the secondary ion mass spectrometry (SIMS) results in Figure 3, compared with the as-implanted dopant profiles prior to annealing, the As and B dopants, after the SIDS process with either MWA or RTA, are found to all pile up and segregate in the vicinity of the NiSi/Si interface. The dopant segregation at the NiSi/Si interface can be explained by the low solid solubility of B and As in nickel silicide [6] and by the negligible diffusion of these dopants in Si at sub-800 °C temperature [18]. The observed larger tail and deviated peak position in the SIMS profile of the As-implanted RTA sample at 377 °C might be attributed to the relatively incomplete transition of NiSi from Ni_2_Si as a result of the retardation of the Ni diffusion due to larger silicide grains induced by As [19]. The deeper distribution of the As compared to that of the B may be attributed to the higher peak concentration of the As and the channel effect of the As during implantation, which may bring a higher doping level of As than of B at a depth of around 120 nm.

The diodes are characterized using capacitance-voltage (C-V) and current-voltage (I-V) measurements. Original C-V data have been re-calculated for the plot of 1/C^2^ *versus* voltage to extract the hole SBH (ϕ_bp_) on the p-type substrate and the electron SBH (ϕ_bn_) on the n-type substrate. The capacitance per unit area of the diodes on n-type substrate is related to the SBH through the following relation [20]:

(1) $$\frac{1}{C_{d}^{2}}=\frac{1}{2q\varepsilon_{Si}N_{D}}\left(\phi_{bn}-\phi_{n}-V-\frac{kT}{q}\right)$$

where *V* is the bias voltage; *N_D_* the net doping concentration of the impurity; and ε_*Si*_ the dielectric constant. The barrier heights were then determined from the intercept of the straight lines on the voltage axis.

**Figure 4:**
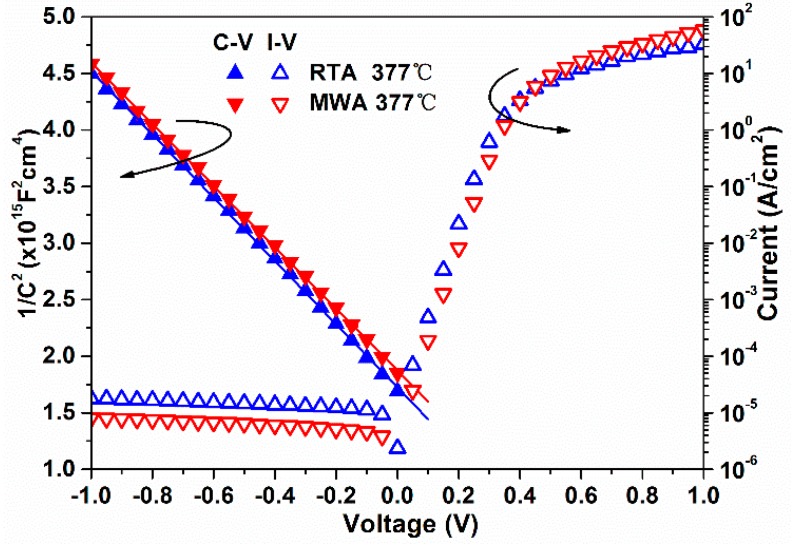
Capacitance-voltage (C-V) and current-voltage (I-V) characteristics of diodes annealed with 600 s on the n-type substrate.

The C-V and I-V characteristics of the produced diodes with B implants into the n-type substrate processed by MWA or RTA at 377 °C for 600 s are shown in Figure 4. The ϕ_bn_ is extracted and found to be 0.94 and 0.87 eV (corresponding ϕ_bp_ are 0.18 and 0.25 eV, since ϕ_bp_ + ϕ_bn_ ≈ 1.12 eV for Si) for the MWA and RTA diodes, respectively. According to the thermionic emission theory, the current of the diode in this paper can be depicted as [21]:

(2) $$J_{n}=A^{*}T^{2}\text{exp}\left(-\frac{q\phi_{bn}}{kT}\right)\left[\text{exp}\left(\frac{qV}{kT}\right)-1\right]$$

where A* is the Richardson’s constant; T the temperature; φ_*bn*_ the barrier height; and V the bias voltage applied. At the same voltage and temperature, the current is the negative exponential function of ϕ_bn_. This can well explain the observation that the leakage current at reverse bias and the forward current when the bias voltage is below 0.35 V of the MWA diode are clearly lower than that of the RTA diode. The higher current of the MWA diode in the over-0.4 V regime should be attributed to a smaller serial resistance. The difference of the serial resistance may come from the contact between the probe and the pad or the substrate and the ground, as well as the diversity that may exist between the silicide formed by two ways of annealing. Nevertheless, the serial resistance is negligible when the voltage ranges from −1.0 V to 0.35 V, as it is too small to share the voltage with the junction. Furthermore, the ideality factor of the MWA diode (1.027) is smaller than that of the RTA diode (1.049), indicating that the MWA diode may have a higher electron SBH, which is consistent with the C-V characteristics.

**Figure 5:**
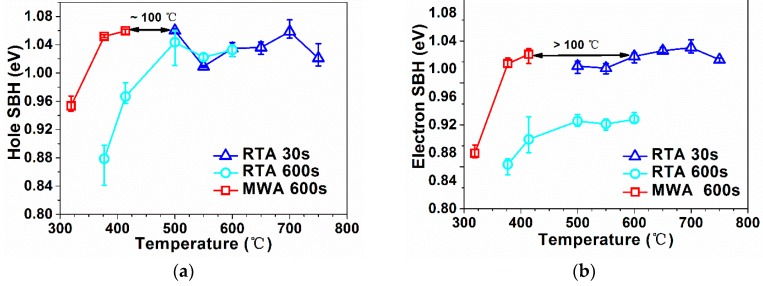
Annealing temperature *versus* (**a**) the hole Schottky barrier height (SBH) (ϕ_bp_) on the p-type substrate; and (**b**) the electron SBH (ϕ_bn_) on the n-type substrate.

The SBH values of the produced diodes are shown in Figure 5 and listed in Table 2. The ideality factors in Table 2 are calculated corresponding to the I-V data around the forward bias voltage of 0.15 V. For both the n-type samples and the p-type samples, the MWA can obtain a SBH tuning effect similar to that of RTP, while the required temperature is ~100 °C lower. The temperature reduction should still be no less than 60 °C, even if the error of the measurement is taken into account. The detailed analysis of the figure is as follows. For the RTA samples with 30-s annealing between 500 and 750 °C, ϕ_bp_ on p-type substrate and ϕ_bn_ on n-type substrate can be tuned to a maximum of 1.06 eV and 0.96 eV, respectively, which is consistent with [6,8]. The observed fluctuation in the SBH at relatively higher temperatures may be related to the roughness and nonuniformity of the poly-NiSi/Si interface and/or possible fluctuations in the activation/deactivation of dopants when the tuning of SBH reaches saturation level. At 500 °C and above, the RTA samples with 600-s annealing show slightly lower SBH levels compared with the RTA samples with 30-s annealing for the n-type substrates, which may be attributed to the NiSi/Si interface degeneration and deactivation of the B dopants at the Si side with longer annealing time (deactivation behavior of B may be different from that of As). For the RTA samples with an As implant, a significant reduction of ϕ_bp_ is found when the temperature decreases from 500 to 414 °C. Similarly, for the RTA samples with a B implant, reduction of ϕ_bn_ is also clearly observed when the temperature decreases from 500 to 414 °C. However, for the MWA diodes annealed at 414 °C, the ϕ_bp_ and ϕ_bn_ is found to be tuned to a maximum of 1.06 eV and 0.95 eV, respectively, proving that the MWA method can tune the SBH to a similar saturated level compared with the RTA method. It is worth noting that the MWA samples annealed as low as 377 °C still demonstrate a sufficiently high ϕ_bp_ of 1.05 eV and ϕ_bn_ of 0.94 eV on the p-type and n-type substrates, respectively.

According to the first-principles calculation, substitutional dopant atoms within the first Si monolayer nearby the NiSi/Si interface can induce electric dipoles across the interface, resulting in the deformation of the energy band and modification of SBH [6,7]. Therefore, it can be inferred that, at 414 °C and below, compared with RTA, MWA is much more effective at substituting Si atoms with dopant atoms at the Si side and hence induce more effective electric dipoles across the NiSi/Si interface, resulting in more SBH tuning with As or B dopants. The capability of tuning SBH between NiSi and Si at a significantly lower temperature using MWA may be ascribed to its selective heating effect [8,9,12] and unique dopant activation mechanisms [10]. For RTA, the activation of dopants is a purely thermally driven process; for MWA, the activation of dopants is a combination of a thermal effect and an additional unique non-thermal microwave effect. The non-thermal effect of MWA in Si may be mainly caused by the rotation and collision of a large amount of dipoles (such as vacancy-interstitial point defects) under the alternating electromagnetic field, which can increase the interaction probability of neighboring dipoles and equivalently reduce the dopant activation energy in Si. Hence, for MWA, the non-thermal effect may enhance the dopant activation at the NiSi/Si interface. As a result, compared with RTA, the effective tuning of SBH can happen at significantly lower bulk-Si temperatures for MWA.

## 4. Conclusions

The MWA method has been successfully used to form NiSi/Si Schottky junction and effectively tune the SBH between NiSi and Si. Compared with conventional RTA method, MWA can obtain saturated electron and hole SBHs at significantly lower temperature. A high hole SBH (ϕ_bp_ = 1.05 eV) and electron SBH (ϕ_bn_ = 0.94 eV), *i.e.*, low electron SBH (ϕ_bn_ = 0.07 eV) and hole SBH (ϕ_bp_ = 0.18 eV), are achieved with MWA at 377 °C on p-type and n-type substrates, respectively. The success of SBH tuning at sub-400 °C via MWA opens the door of formation of metal source/drain and reduction of silicide contact resistance during the back-end of process (BEOL) as well as application of MWA in future monolithic 3D sequential integration where low-temperature formation of source/drain and contacts is of high importance. More comprehensive research on the metal/semiconductor contacts treated via MWA will be subsequently conducted using more test structures including circular Transmission Line Measurement (TLM) [22] and Schottky barrier MOSFETs.

## Figures and Tables

**Table 1:** Annealing temperature and duration for both microwave annealing (MWA) and rapid thermal annealing (RTA) techniques. (The corresponding microwave power for 319 °C, 377 °C, and 414 °C MWA are 2100 W, 2800 W, and 3500 W, respectively.

Implanted Ions/Substrate	Arsenic/p-Type			Boron/n-Type		
Annealing technique	MWA	RTA	RTA	MWA	RTA	RTA
Time (s)	600	600	30	600	600	30
Temperature (°C)	319	377	500	319	377	500
	377	414	550	377	414	550
	414	500	600	414	500	600
	-	550	650	-	550	650
	-	600	700	-	600	700
	-	-	750	-	-	750

**Table 2:** Effective SBH and ideality factor of each annealing condition.

Annealing Technique	Time (s)	Temperature (°C)	Implanted Ions/Substrate			
			Arsenic/p-Type		Boron/n-Type	
			ϕ_bp_ (eV)	Ideality Factor (@ −0.15 V)	ϕ_bn_ (eV)	Ideality Factor (@ 0.15 V)
MWA	600	319	0.96	1.077	0.85	1.021
		377	1.05	1.125	0.94	1.027
		414	1.06	1.074	0.95	1.025
RTA	600	377	0.88	1.024	0.86	1.049
		414	0.97	1.092	0.90	1.050
		500	1.04	1.063	0.93	1.056
		550	1.02	1.118	0.92	1.034
		600	1.03	1.065	0.93	1.037
	30	500	1.06	1.012	0.94	1.025
		550	1.01	1.029	0.93	1.018
		600	1.03	1.059	0.95	1.025
		650	1.04	1.043	0.95	1.043
		700	1.06	1.093	0.95	1.060
		750	1.02	1.121	0.94	1.005

## Appendix A

The accuracy of temperature measurement is crucial in this experiment. The MWA facility uses an infrared pyrometer to read the temperature of the sample, while the RTP system uses a regular thermal couple. Hence, the key uncertainty will be the MWA’s temperature profile. To obtain the deviation of the temperature measurement of the MWA process, a calibration system was built. As shown in Figure A1, the same model of infrared pyrometer as the one used in the MWA facility was used, and a regular S-type thermal couple was induced as a reference. The samples (n-type or p-type) as described in the main text were put on a heat unit with their backside facing the infrared pyrometer located above. The heat unit consisted of a thermal plate and three square-shaped silicon wafers with thicknesses of 0.5 mm. The upper two wafers were as close as possible except for only a narrow slit for the mounting of the thermal couple to obtain optimum heat conduction from the heat plate to the sample. A successful contact between the thermal couple and the surface of the sample was acquired, attributed to the equivalence of the diameter of the thermal couple and the thickness of the silicon wafer.

The temperature profile of the thermal couple and the pyrometer were recorded and are shown in Figure A2. The calibration started from around 170 °C and ended at a similar temperature after heating and a naturally cooling process. A stable ~400 °C (a temperature level similar to the highest group of MWA in the main text) platform was applied in the middle of the heating process. During the whole calibration work, the pyrometer read a lower temperature than the thermal couple. The maximum temperature differences of these two measurement were 43 °C and 30 °C for the n-type and p-type samples, respectively. Considering that the environment temperature of the calibration system should not be higher than that of the MWA facility, the difference of the environment between the MWA equipment and the calibration system will not enlarge the deviation of the temperature measurement. Therefore, the MWA’s temperature profile is underestimated, and the error should not surpass 43 °C and 30 °C for the n-type and p-type samples, respectively.

As the thermal couple was put next to the back side of the sample, a question may be raised about the calibration system: Will an obvious discrepancy in temperature exist between the two sides of the sample? A simple simulation using COMSOL Multiphysics (Stockholm, Sweden) was carried out to study the temperature difference that may exist between the two sides of the wafer. A four-inch silicon wafer was set in the model with the upper side defined as nickel and the backside defined as silicon. A heat flux of 12,000 W/m^2^ was applied to the upper side as the heat source of the wafer. (This setup is different from what actually happened in the MWA process because the whole sample was heated by the microwave simultaneously; however, this setup can give out the largest temperature diversity that may exist between the two sides). The wafer was located in the middle of a chamber and an air flow was set to simulate the nitrogen flow in the real MWA chamber. Heat radiation of the wafer was also taken into consideration, with different emissivities for the two sides of the wafer (0.3 for the nickel and 0.7 for the silicon).

The overall temperature of the simulation of both the chamber and the wafer is shown in Figure A3. The highest temperature is located at the wafer and its vicinity. The zoomed cross-sectional temperature profile and the detailed value *versus* the depth from the surface of the wafer are shown in Figure A4. The temperature varies less than 0.1 °C throughout the thickness of the wafer. Hence, there will not be obvious disparity between the two sides of the wafer according to the simulation. The measurement of the backside of the wafer can be regarded as a valid way to monitor the temperature of the reacting zone in the sample.
